# Bis(3,5-dimethyl-1*H*-pyrazole-κ*N* ^2^)(pyridine-2,6-dicarboxyl­ato-κ^3^ *O* ^2^,*N*,*O* ^6^)copper(II)

**DOI:** 10.1107/S1600536809004577

**Published:** 2009-02-18

**Authors:** Yuan-Yuan Lin, Yan-Ping Yu, Bing-Xin Liu, Liang-Jun Zhang

**Affiliations:** a Department of Chemistry, Shanghai University, Shanghai 200444, People’s Republic of China; b Department of Petroleum and Chemical Industry, Guangxi Vocational and Technical Institute of Industry, People’s Republic of China

## Abstract

In the crystal structure of the title compound, [Cu(C_7_H_3_NO_4_)(C_5_H_8_N_2_)_2_], the Cu^II^ cation assumes a distorted trigonal–bipyramidal coordination geometry formed by a pyridine-2,6-dicarboxyl­ate dianion and two 3,5-dimethyl-1*H*-pyrazole mol­ecules. N—H⋯O hydrogen bonding is present in the crystal structure.

## Related literature

For general background, see: Haanstra *et al.* (1990 ▶); Mukherjee (2000 ▶).
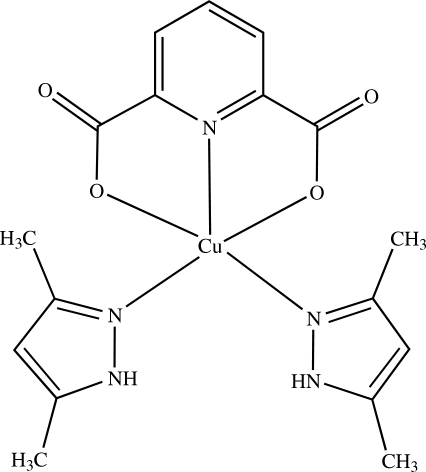


## Experimental

### 

#### Crystal data

[Cu(C_7_H_3_NO_4_)(C_5_H_8_N_2_)_2_]
*M* *_r_* = 420.91
Triclinic, 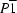

*a* = 8.4572 (12) Å
*b* = 8.5083 (12) Å
*c* = 13.942 (2) Å
α = 72.986 (2)°
β = 85.500 (2)°
γ = 66.760 (2)°
*V* = 880.7 (2) Å^3^
*Z* = 2
Mo *K*α radiation
μ = 1.28 mm^−1^
*T* = 295 K
0.23 × 0.15 × 0.13 mm

#### Data collection

Bruker APEX CCD diffractometer
Absorption correction: multi-scan (*SADABS*; Sheldrick, 1996 ▶) *T*_min_ = 0.775, *T*_max_ = 0.845
4570 measured reflections
3036 independent reflections
2497 reflections with *I* > 2σ(*I*)
*R*_int_ = 0.020

#### Refinement

*R*[*F* ^2^ > 2σ(*F* ^2^)] = 0.044
*wR*(*F* ^2^) = 0.106
*S* = 1.05
3036 reflections
248 parameters
H-atom parameters constrained
Δρ_max_ = 0.67 e Å^−3^
Δρ_min_ = −0.55 e Å^−3^

### 

Data collection: *SMART* (Bruker, 2004 ▶); cell refinement: *SAINT* (Bruker, 2004 ▶); data reduction: *SAINT*; program(s) used to solve structure: *SIR92* (Altomare *et al.*, 1993 ▶); program(s) used to refine structure: *SHELXL97* (Sheldrick, 2008 ▶); molecular graphics: *ORTEP-3 for Windows* (Farrugia, 1997 ▶); software used to prepare material for publication: *WinGX* (Farrugia, 1999 ▶).

## Supplementary Material

Crystal structure: contains datablocks I, global. DOI: 10.1107/S1600536809004577/xu2458sup1.cif

Structure factors: contains datablocks I. DOI: 10.1107/S1600536809004577/xu2458Isup2.hkl

Additional supplementary materials:  crystallographic information; 3D view; checkCIF report

## Figures and Tables

**Table 1:** Selected bond lengths (Å)

Cu—N11	1.917 (3)
Cu—N21	2.172 (3)
Cu—N31	1.994 (3)
Cu—O11	2.025 (2)
Cu—O13	2.006 (2)

**Table 2:** Hydrogen-bond geometry (Å, °)

*D*—H⋯*A*	*D*—H	H⋯*A*	*D*⋯*A*	*D*—H⋯*A*
N22—H22*A*⋯O14^i^	0.86	2.10	2.888 (4)	151
N32—H32*A*⋯O12^ii^	0.86	2.06	2.860 (4)	155

Symmetry codes: (i) 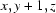; (ii) 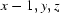.

**Fig. 1.**
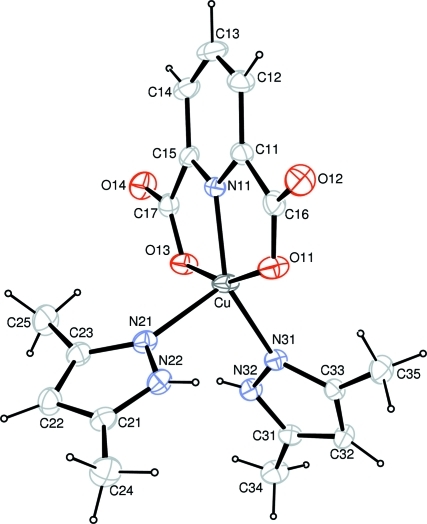
The molecular structure of (I) with 30% probability displacement ellipsoids.

**Fig. 2.**
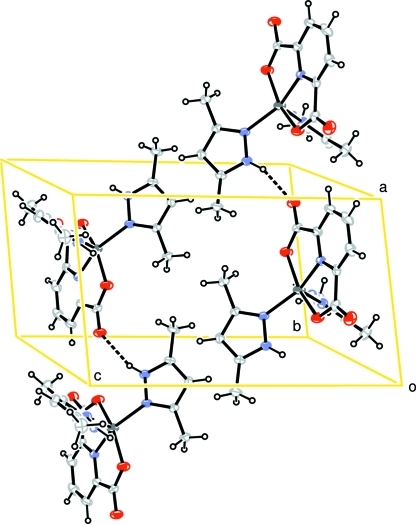
The unit cell packing diagram showing hydrogen bonding (dashed lines).

## supplementary crystallographic information

### Comment

Complexes with pyrazole-based ligands are a frequent subject of chemical investigations giving an opportunity for a better understanding the relationship between the structure and the activity of the active site of metalloproteins (Haanstra *et al.*, 1990). Nowadays, attention is paid to the design of various pyrazole ligands with special structural properties to fulfill the specific stereochemical requirements of a particular metal-binding site (Mukherjee, 2000). In our systematic studies on transition metal complexes with the pyrazole derivatives, the title compound was prepared and its X-ray structure is presented here.

The molecular structure of the title compound is shown in Fig. 1. The compound assumes a distorted triangular bipyramid coordination geometry (Table 1), formed by a pyridine-2,6-dicarboxylate dianion and two 3,5-dimethyl-1-*H*-pyrazole molecules. Tridentate ligand pyridine-2,6-dicarboxylate dianion chelates to the Cu atom by a N atom of pyridine ring and two O atoms of carboxyl groups with a meridional configuration. Monodentate ligand 3,5-dimethyl-1-*H*-pyrazole coordinated to the Cu atom by N atoms of pyrazole rings with the 1.917 (3) Å and 1.994 (3) Å of Cu—N bound distance. The adjacent molecules are linked together *via* N—H···O hydrogen bonding (Table 2) between carboxy groups of pyridine-2,6-dicarboxylate dianion and uncoordinated N atom of 3,5-dimethyl-1-*H*-pyrazoleto, forming the supra-molecular structure (Fig. 2).

### Experimental

An ethanol–water solution (1:1, 20 ml) containing 1-carboxamide-3,5-dimethylpyrazole (0.14 g, 1 mmol) and CuCl_2_.2H_2_O (0.17 g, 1 mmol) was mixed with an aqueous solution (10 ml) of pyridine-2,3-dicarboxylic acid (0.17 g, 1 mmol) and NaOH (0.08 g, 2 mmol). The mixture was refluxed for 6 h. After cooling to room temperature the solution was filtered. Single crystals were obtained from the filtrate after 3 d.

### Refinement

Methyl H were placed in calculated positions with C—H = 0.96 Å and torsion angles were refined to fit the electron density, *U*_iso_(H) = 1.5*U*_eq_(C). Other H atoms were placed in calculated positions with C—H = 0.93 Å and N—H = 0.86 Å, and refined in riding mode with *U*_iso_(H) = 1.2*U*_eq_(C,N).

### Crystal data

**Table:**

[Cu(C_7_H_3_NO_4_)(C_5_H_8_N_2_)_2_]	*Z* = 2
*M**_r_* = 420.91	*F*(000) = 434
Triclinic, *P*1	*D*_x_ = 1.587 Mg m^−^^3^
Hall symbol: -P 1	Mo *K*α radiation, λ = 0.71073 Å
*a* = 8.4572 (12) Å	Cell parameters from 2980 reflections
*b* = 8.5083 (12) Å	θ = 2.0–25.0°
*c* = 13.942 (2) Å	µ = 1.27 mm^−^^1^
α = 72.986 (2)°	*T* = 295 K
β = 85.500 (2)°	Prism, blue
γ = 66.760 (2)°	0.23 × 0.15 × 0.13 mm
*V* = 880.7 (2) Å^3^	

### Data collection

**Table:**

Bruker APEX CCD diffractometer	3036 independent reflections
Radiation source: fine-focus sealed tube	2497 reflections with *I* > 2σ(*I*)
graphite	*R*_int_ = 0.020
φ and ω scans	θ_max_ = 25.0°, θ_min_ = 2.6°
Absorption correction: multi-scan (*SADABS*; Sheldrick, 1996)	*h* = −7→10
*T*_min_ = 0.775, *T*_max_ = 0.845	*k* = −8→10
4570 measured reflections	*l* = −16→14

### Refinement

**Table:**

Refinement on *F*^2^	Primary atom site location: structure-invariant direct methods
Least-squares matrix: full	Secondary atom site location: difference Fourier map
*R*[*F*^2^ > 2σ(*F*^2^)] = 0.044	Hydrogen site location: inferred from neighbouring sites
*wR*(*F*^2^) = 0.106	H-atom parameters constrained
*S* = 1.05	*w* = 1/[σ^2^(*F*_o_^2^) + (0.044*P*)^2^ + 0.8468*P*] where *P* = (*F*_o_^2^ + 2*F*_c_^2^)/3
3036 reflections	(Δ/σ)_max_ < 0.001
248 parameters	Δρ_max_ = 0.67 e Å^−^^3^
0 restraints	Δρ_min_ = −0.55 e Å^−^^3^

### Special details

**Table:**

Geometry. All e.s.d.'s (except the e.s.d. in the dihedral angle between two l.s. planes) are estimated using the full covariance matrix. The cell e.s.d.'s are taken into account individually in the estimation of e.s.d.'s in distances, angles and torsion angles; correlations between e.s.d.'s in cell parameters are only used when they are defined by crystal symmetry. An approximate (isotropic) treatment of cell e.s.d.'s is used for estimating e.s.d.'s involving l.s. planes.
Refinement. Refinement of *F*^2^ against ALL reflections. The weighted *R*-factor *wR* and goodness of fit *S* are based on *F*^2^, conventional *R*-factors *R* are based on *F*, with *F* set to zero for negative *F*^2^. The threshold expression of *F*^2^ > σ(*F*^2^) is used only for calculating *R*-factors(gt) *etc*. and is not relevant to the choice of reflections for refinement. *R*-factors based on *F*^2^ are statistically about twice as large as those based on *F*, and *R*- factors based on ALL data will be even larger.

### Fractional atomic coordinates and isotropic or equivalent isotropic displacement parameters (Å^2^)

**Table:**

	*x*	*y*	*z*	*U*_iso_*/*U*_eq_	
Cu	0.48595 (5)	0.08560 (6)	0.27695 (4)	0.03571 (17)	
O11	0.6674 (3)	0.1852 (3)	0.2773 (2)	0.0441 (7)	
O12	0.9462 (3)	0.1267 (4)	0.2534 (2)	0.0532 (8)	
O13	0.3807 (3)	−0.0884 (3)	0.2770 (2)	0.0422 (6)	
O14	0.4427 (4)	−0.3530 (4)	0.2516 (2)	0.0509 (7)	
N11	0.6858 (3)	−0.1038 (4)	0.2513 (2)	0.0293 (6)	
N21	0.3623 (4)	0.2984 (4)	0.1424 (2)	0.0337 (7)	
N22	0.3694 (4)	0.4602 (4)	0.1294 (2)	0.0350 (7)	
H22A	0.4223	0.4823	0.1709	0.042*	
N31	0.3204 (3)	0.1863 (4)	0.3744 (2)	0.0314 (7)	
N32	0.1637 (3)	0.1746 (4)	0.3799 (2)	0.0331 (7)	
H32A	0.1283	0.1307	0.3419	0.040*	
C11	0.8379 (4)	−0.0898 (5)	0.2490 (3)	0.0347 (8)	
C12	0.9861 (5)	−0.2312 (5)	0.2403 (3)	0.0443 (10)	
H12	1.0932	−0.2237	0.2375	0.053*	
C13	0.9699 (5)	−0.3839 (5)	0.2359 (3)	0.0512 (11)	
H13	1.0683	−0.4808	0.2303	0.061*	
C14	0.8112 (5)	−0.3972 (5)	0.2396 (3)	0.0437 (10)	
H14	0.8016	−0.5008	0.2365	0.052*	
C15	0.6684 (4)	−0.2511 (4)	0.2481 (2)	0.0328 (8)	
C16	0.8196 (4)	0.0886 (5)	0.2600 (3)	0.0363 (8)	
C17	0.4818 (5)	−0.2331 (5)	0.2587 (3)	0.0350 (8)	
C21	0.2857 (5)	0.5808 (5)	0.0456 (3)	0.0375 (8)	
C22	0.2196 (5)	0.4948 (5)	0.0004 (3)	0.0424 (9)	
H22	0.1546	0.5431	−0.0597	0.051*	
C23	0.2699 (5)	0.3211 (5)	0.0628 (3)	0.0377 (9)	
C24	0.2736 (6)	0.7685 (5)	0.0153 (3)	0.0524 (11)	
H24A	0.2520	0.8126	0.0731	0.079*	
H24B	0.1810	0.8414	−0.0340	0.079*	
H24C	0.3798	0.7723	−0.0126	0.079*	
C25	0.2362 (6)	0.1690 (6)	0.0483 (3)	0.0555 (11)	
H25A	0.1908	0.1150	0.1082	0.083*	
H25B	0.3418	0.0819	0.0341	0.083*	
H25C	0.1543	0.2128	−0.0069	0.083*	
C31	0.0715 (4)	0.2404 (5)	0.4523 (3)	0.0344 (8)	
C32	0.1727 (4)	0.2944 (5)	0.4958 (3)	0.0361 (8)	
H32	0.1444	0.3447	0.5489	0.043*	
C33	0.3257 (4)	0.2602 (4)	0.4457 (2)	0.0300 (8)	
C34	−0.1051 (4)	0.2448 (6)	0.4742 (3)	0.0463 (10)	
H34A	−0.1235	0.1620	0.4466	0.069*	
H34B	−0.1884	0.3630	0.4445	0.069*	
H34C	−0.1174	0.2121	0.5455	0.069*	
C35	0.4794 (5)	0.2936 (6)	0.4643 (3)	0.0458 (10)	
H35A	0.5760	0.1819	0.4864	0.069*	
H35B	0.4554	0.3548	0.5150	0.069*	
H35C	0.5057	0.3658	0.4033	0.069*	

### Atomic displacement parameters (Å^2^)

**Table:**

	*U*^11^	*U*^22^	*U*^33^	*U*^12^	*U*^13^	*U*^23^
Cu	0.0240 (2)	0.0284 (3)	0.0603 (3)	−0.01015 (18)	0.00311 (19)	−0.0211 (2)
O11	0.0298 (13)	0.0415 (15)	0.0719 (19)	−0.0164 (12)	0.0073 (13)	−0.0298 (14)
O12	0.0338 (15)	0.074 (2)	0.070 (2)	−0.0317 (14)	0.0095 (13)	−0.0341 (16)
O13	0.0317 (13)	0.0328 (14)	0.0679 (18)	−0.0128 (11)	−0.0016 (12)	−0.0216 (13)
O14	0.0624 (18)	0.0405 (16)	0.0659 (19)	−0.0304 (14)	0.0011 (14)	−0.0235 (14)
N11	0.0264 (14)	0.0292 (15)	0.0309 (15)	−0.0084 (12)	0.0004 (12)	−0.0099 (12)
N21	0.0346 (16)	0.0286 (16)	0.0452 (18)	−0.0157 (13)	0.0031 (14)	−0.0168 (13)
N22	0.0402 (17)	0.0316 (16)	0.0422 (18)	−0.0195 (14)	0.0032 (14)	−0.0159 (14)
N31	0.0228 (14)	0.0334 (16)	0.0435 (17)	−0.0137 (12)	0.0031 (12)	−0.0154 (13)
N32	0.0275 (15)	0.0378 (17)	0.0418 (18)	−0.0177 (13)	0.0037 (13)	−0.0163 (14)
C11	0.0294 (18)	0.041 (2)	0.0299 (19)	−0.0110 (16)	0.0026 (15)	−0.0092 (16)
C12	0.0291 (19)	0.052 (3)	0.044 (2)	−0.0086 (18)	0.0083 (17)	−0.0135 (19)
C13	0.046 (2)	0.040 (2)	0.049 (2)	0.0004 (19)	0.0159 (19)	−0.0142 (19)
C14	0.054 (2)	0.030 (2)	0.042 (2)	−0.0102 (18)	0.0095 (19)	−0.0140 (17)
C15	0.042 (2)	0.0288 (19)	0.0280 (18)	−0.0117 (16)	0.0020 (15)	−0.0115 (15)
C16	0.0307 (19)	0.045 (2)	0.038 (2)	−0.0175 (17)	−0.0014 (16)	−0.0151 (17)
C17	0.044 (2)	0.0299 (19)	0.033 (2)	−0.0163 (17)	−0.0036 (16)	−0.0080 (15)
C21	0.042 (2)	0.033 (2)	0.040 (2)	−0.0161 (17)	0.0095 (17)	−0.0135 (17)
C22	0.051 (2)	0.041 (2)	0.040 (2)	−0.0217 (19)	−0.0025 (18)	−0.0133 (18)
C23	0.042 (2)	0.034 (2)	0.042 (2)	−0.0176 (17)	0.0028 (17)	−0.0149 (17)
C24	0.072 (3)	0.034 (2)	0.054 (3)	−0.026 (2)	0.004 (2)	−0.0104 (19)
C25	0.072 (3)	0.047 (3)	0.063 (3)	−0.032 (2)	−0.010 (2)	−0.022 (2)
C31	0.0277 (18)	0.034 (2)	0.041 (2)	−0.0130 (16)	0.0045 (15)	−0.0096 (16)
C32	0.039 (2)	0.044 (2)	0.033 (2)	−0.0211 (18)	0.0062 (16)	−0.0168 (16)
C33	0.0290 (18)	0.0294 (18)	0.0334 (19)	−0.0135 (15)	−0.0015 (15)	−0.0077 (15)
C34	0.032 (2)	0.054 (3)	0.061 (3)	−0.0224 (19)	0.0146 (18)	−0.022 (2)
C35	0.038 (2)	0.060 (3)	0.053 (2)	−0.028 (2)	0.0018 (18)	−0.025 (2)

### Geometric parameters (Å, °)

**Table:**

Cu—N11	1.917 (3)	C14—C15	1.377 (5)
Cu—N21	2.172 (3)	C14—H14	0.9300
Cu—N31	1.994 (3)	C15—C17	1.523 (5)
Cu—O11	2.025 (2)	C21—C22	1.376 (5)
Cu—O13	2.006 (2)	C21—C24	1.491 (5)
O11—C16	1.274 (4)	C22—C23	1.390 (5)
O12—C16	1.225 (4)	C22—H22	0.9300
O13—C17	1.277 (4)	C23—C25	1.500 (5)
O14—C17	1.221 (4)	C24—H24A	0.9600
N11—C15	1.332 (4)	C24—H24B	0.9600
N11—C11	1.334 (4)	C24—H24C	0.9600
N21—C23	1.331 (4)	C25—H25A	0.9600
N21—N22	1.360 (4)	C25—H25B	0.9600
N22—C21	1.332 (5)	C25—H25C	0.9600
N22—H22A	0.8600	C31—C32	1.366 (5)
N31—C33	1.335 (4)	C31—C34	1.489 (5)
N31—N32	1.362 (3)	C32—C33	1.388 (5)
N32—C31	1.343 (4)	C32—H32	0.9300
N32—H32A	0.8600	C33—C35	1.492 (4)
C11—C12	1.380 (5)	C34—H34A	0.9600
C11—C16	1.516 (5)	C34—H34B	0.9600
C12—C13	1.378 (6)	C34—H34C	0.9600
C12—H12	0.9300	C35—H35A	0.9600
C13—C14	1.387 (6)	C35—H35B	0.9600
C13—H13	0.9300	C35—H35C	0.9600
			
N11—Cu—N31	149.17 (12)	O14—C17—O13	126.4 (4)
N11—Cu—O13	80.43 (11)	O14—C17—C15	119.8 (3)
N31—Cu—O13	92.70 (11)	O13—C17—C15	113.7 (3)
N11—Cu—O11	79.88 (11)	N22—C21—C22	106.1 (3)
N31—Cu—O11	102.35 (10)	N22—C21—C24	122.8 (3)
O13—Cu—O11	159.96 (10)	C22—C21—C24	131.1 (4)
N11—Cu—N21	113.60 (11)	C21—C22—C23	105.9 (3)
N31—Cu—N21	97.19 (11)	C21—C22—H22	127.0
O13—Cu—N21	101.01 (10)	C23—C22—H22	127.0
O11—Cu—N21	90.27 (11)	N21—C23—C22	110.8 (3)
C16—O11—Cu	115.7 (2)	N21—C23—C25	120.8 (3)
C17—O13—Cu	116.0 (2)	C22—C23—C25	128.4 (3)
C15—N11—C11	123.2 (3)	C21—C24—H24A	109.5
C15—N11—Cu	117.9 (2)	C21—C24—H24B	109.5
C11—N11—Cu	118.4 (2)	H24A—C24—H24B	109.5
C23—N21—N22	104.5 (3)	C21—C24—H24C	109.5
C23—N21—Cu	137.2 (2)	H24A—C24—H24C	109.5
N22—N21—Cu	118.3 (2)	H24B—C24—H24C	109.5
C21—N22—N21	112.7 (3)	C23—C25—H25A	109.5
C21—N22—H22A	123.6	C23—C25—H25B	109.5
N21—N22—H22A	123.6	H25A—C25—H25B	109.5
C33—N31—N32	105.7 (3)	C23—C25—H25C	109.5
C33—N31—Cu	135.3 (2)	H25A—C25—H25C	109.5
N32—N31—Cu	118.9 (2)	H25B—C25—H25C	109.5
C31—N32—N31	111.4 (3)	N32—C31—C32	106.3 (3)
C31—N32—H32A	124.3	N32—C31—C34	122.6 (3)
N31—N32—H32A	124.3	C32—C31—C34	131.1 (3)
N11—C11—C12	119.7 (3)	C31—C32—C33	107.0 (3)
N11—C11—C16	111.7 (3)	C31—C32—H32	126.5
C12—C11—C16	128.6 (3)	C33—C32—H32	126.5
C13—C12—C11	117.7 (4)	N31—C33—C32	109.5 (3)
C13—C12—H12	121.1	N31—C33—C35	121.9 (3)
C11—C12—H12	121.1	C32—C33—C35	128.5 (3)
C12—C13—C14	121.9 (3)	C31—C34—H34A	109.5
C12—C13—H13	119.0	C31—C34—H34B	109.5
C14—C13—H13	119.0	H34A—C34—H34B	109.5
C15—C14—C13	117.4 (4)	C31—C34—H34C	109.5
C15—C14—H14	121.3	H34A—C34—H34C	109.5
C13—C14—H14	121.3	H34B—C34—H34C	109.5
N11—C15—C14	120.0 (3)	C33—C35—H35A	109.5
N11—C15—C17	111.8 (3)	C33—C35—H35B	109.5
C14—C15—C17	128.2 (3)	H35A—C35—H35B	109.5
O12—C16—O11	126.2 (3)	C33—C35—H35C	109.5
O12—C16—C11	119.7 (3)	H35A—C35—H35C	109.5
O11—C16—C11	114.1 (3)	H35B—C35—H35C	109.5

### Hydrogen-bond geometry (Å, °)

**Table:**

*D*—H···*A*	*D*—H	H···*A*	*D*···*A*	*D*—H···*A*
N22—H22A···O14^i^	0.86	2.10	2.888 (4)	151
N32—H32A···O12^ii^	0.86	2.06	2.860 (4)	155

Symmetry codes: (i) *x*, *y*+1, *z*; (ii) *x*−1, *y*, *z*.
